# Supplementary material for: Mental health services accessibility for women and men: A protocol for a systematic review and meta-analysis
Source: PLoS One. 2022 Nov 16;17(11):e0277661. doi: 10.1371/journal.pone.0277661 (PMC9668153; doi:10.1371/journal.pone.0277661)
Supplement: Attachment — Submitted filename: Response to Reviewers.docx [file pone.0277661.s002.docx]

Response to Reviewers

Dear Reviewer,

We appreciate contributions to our protocol article. We hope that this article is accepted and that it can contribute to your journal that has a great international impact. All points suggested were accepted and corrected in the article file.

The following points were corrected and answered:

- 1- The protocol registration date was corrected;
- 2- As the systematic review project was already underway, only a partial result was included in the protocol article, as the systematic review article is yet to be submitted. If partial data is not necessary, please inform that they will be removed from the protocol article.
- 3- The sentence was corrected using PRISMA for the protocol. The issue of using PRISMA as methodological guidance was modified in the methodology, we modified the sentence to "The protocol is being reported and directed according to the Declaration of Preferred Reporting Items for Systematic Review and Meta-Analysis Protocols (PRISMA-P) " according to the suggested methodology: Sarkis-Onofre, R., Catalá-López, F., Aromataris, E. et al. How to properly use the PRISMA Statement. System Rev 10, 117 (2021). <https://doi.org/10.1186/s13643-021-01671-z>
- 4- The LILACS database was modified in the figure of the flowchart, since the database used was CINAHL;
- 5- The descriptions in tables 1 and 2 have been corrected;
- 6- As the protocol involves observational studies, the acronym PICO was changed to PECO, in which the studies involve exposure and non-intervention;
- 7- A more detailed description of the study eligibility criteria has been included;
- 8- Regarding the concepts of accessibility questioned in the introduction, accessibility is a very broad concept with several aspects, taking into account this, it was worked on in the systematic review as access in general, resulting in the arrival of the woman or the man in the mental health service;
- 9- There were no language, country or year restrictions regarding the studies included in the protocol/systematic review. This information was adjusted in the methodology;
- 10- Regarding missing data in data extraction, when the data on the prevalence of accessibility to mental health services is absent, if they have data available for the calculation, the authors will perform the prevalence calculation. Regarding the evaluation of the included studies, studies with a representative sample will be considered. Studies will be assessed for bias by the Jonna Brigs checklist for cross-sectional studies;
- 11- The term has been changed to "Risk of bias and quality assessment" as it better reproduces the objective of the Joanna Briggs assessment, which includes an assessment of the methodological quality of a study and determining the extent to which a study addressed the possibility of bias in its design, conduct and analysis;
- 12- They were included as a study in the discussion.

In response to the editor:

- The sentence was corrected using PRISMA for the protocol. The issue of using PRISMA as methodological guidance was modified in the methodology, we modified the sentence to "The protocol is being reported and directed according to the Declaration of Preferred Reporting Items for Systematic Review and Meta-Analysis Protocols (PRISMA-P) " according to the suggested methodology: Sarkis-Onofre, R., Catalá-López, F., Aromataris, E. et al. How to properly use the PRISMA Statement. System Rev 10, 117 (2021). <https://doi.org/10.1186/s13643-021-01671-z>
- The editor suggested review on the decision to use fixed or random models. We decided to use random effects, due to the variety of studies that we will find and the possibility of including few studies in a meta-analysis. In this method, the variability of studies is considered. In addition, in this method, the weight of smaller studies is greater when compared to fixed-effect studies.
