# Supplementary material for: Mental health services accessibility for women and men: A protocol for a systematic review and meta-analysis
Source: PLoS One. 2022 Nov 16;17(11):e0277661. doi: 10.1371/journal.pone.0277661 (PMC9668153; doi:10.1371/journal.pone.0277661)
Supplement: Attachment — Submitted filename: Response to Reviewers.docx [file pone.0277661.s003.docx]

Response to Reviewers

Dear Reviewer,

We appreciate contributions to our protocol article. We hope that this article is accepted and that it can contribute to your journal that has a great international impact. All points suggested were accepted and corrected in the article file.

The following points were corrected and answered:

- 1- we have included a sentence in the methods that indicates that there are no results in this study, and that there no other articles submitted or published with the results of this research;
- 2- The statement in the abstract was corrected.
- 3- The phrase "The protocol is being reported and directed in accordance with the Preferred Reporting Items for Systematic Review and Meta-Analysis Protocols (PRISMA-P)" was transferred to the topic "Protocol and register".
- 4- The description of table 2 was corrected.;
- 5- the requested answers were included in the methods section, in the item "inclusion criteria";
- 6-The exclusion criteria have been corrected;
- 7- The suggested limitations have been added.
